# Molecular Mechanisms of Neural Circuit Development and Regeneration

**DOI:** 10.3390/ijms22094593

**Published:** 2021-04-27

**Authors:** Lieve Moons, Lies De Groef

**Affiliations:** 1 Neural Circuit Development and Regeneration Research Group, Biology Department, University of Leuven, Naamsestraat 61 Box 2464, 3000 Leuven, Belgium; lieve.moons@kuleuven.be; 2 Leuven Brain Institute, 3000 Leuven, Belgium

The human brain contains 86 billion neurons [1], which are connected to thousands of other neurons and thereby function in a complex spatiotemporal organization that forms the physiological and biological basis of cognition. Understanding how these neural circuits are formed and what is needed for them to be repaired is one of the biggest challenges in modern science. Studies in humans and different animal models have helped to unravel the principles of neural circuit formation yet have also pointed out striking differences in the regenerative capacity of the central nervous system (CNS) among species, with the mammalian (human) adult CNS being incapable of neurorepair. Further insights into the gene networks, molecular players, and (sub)cellular entities responsible for neural circuit formation are still urgently needed and may propel the search for neuroprotective and neuroreparative strategies to treat neurodevelopmental and neurodegenerative conditions.

In this Special Issue, the roles of Tau [2], heme oxygenase [3], and calsequestrin [4] in axonal regeneration, circuit formation, and synaptic plasticity are revisited. Tam et al. also report on their search for cerebellar markers that may propel research into the (mal)development and (dys)function of the cerebellum [5]. Furthermore, novel research on the molecular and electrophysiological mechanisms by which GABAergic input regulates adult neurogenesis in the dentate gyrus is presented [6], as well as on the role of developmentally regulated GTP-binding protein 2 (DRG2) as a modulator of the dopaminergic system [7]. The molecular mechanisms of CNS circuit formation are further explored in review manuscripts that concentrate on different developmental stages: Hanswijk et al. [8] focus on the serotonergic system in brain development, Kim and Park on activity-dependent neurogenesis in adulthood [9], and Uyeda and Muramatsu [10] on axonal regeneration and remyelination during brain repair.

From the early ages of neuroscience, it is has become apparent that the basic principles for building the CNS are often common for development and repair. Understanding how neural circuit formation is orchestrated in the developing CNS, and how regenerative capacities were lost over the course of evolution, may help researchers to design pro-regenerative therapies in which developmental programs are recapitulated to regenerate adult neural circuits. Comparative neuroscience research may thus hold the key to future restorative therapies for CNS injury and disease in humans. Similarly, we may learn by studying the parallels and contrasts of the CNS versus the regeneration-competent peripheral nervous system. In this respect, three publications in this Special Issue are of particular interest. First, Pereiro et al. lay the foundations for understanding the role of RNA-binding protein with multiple splicing (RBPMS) in axonal and dendritic regeneration by studying it in various stages of development and different types of injury [11]. Second, Yusifov and colleagues present novel research on the role of the primary cilium in peripheral nervous system formation and describe differences compared to the ciliation of neurons in the CNS [12]. Finally, Petrova et al. touch upon the role of axonal organelles as hubs organizing the machinery for axonal outgrowth and growth cone formation. They provide a comprehensive discussion of the role of organelles as highly dynamic and interconnected molecular platforms with a central role in developmental axon growth, and describe how these organelles can be targeted to boost axon regeneration [13].

The latter concept, of growth-regulated changes in protein synthesis and turnover, cargo translocation, and energy supply in the different compartments of a neuron—in particular in the axon and its growth cone—is also the topic of the final two articles in this Special Issue. Relatively new areas of investigation in the neuroregeneration field are presented by Corradi and Baudet [14] and Spead and Poulain [15], who review the latest insights into the role of miRNAs in regulating local translation in the presynaptic axonal compartment and into the molecular mechanisms underlying transaxonal signaling during neural circuit formation, respectively.

This Special Issue offers a view on current neuroscience research into neural circuit development and regeneration in the CNS. Via six original papers and eight review manuscripts, it sketches the ongoing research efforts to disentangle the molecular and cellular programs directing neural circuit development and repair. Together, and interacting with each other, these two research areas (i.e., developmental and regenerative neurobiology) may lead to future neurorestorative therapies that are so desperately needed to relieve the burden of neurodegenerative and neurodevelopmental diseases.

## Data Availability

Not applicable.
